# Assessing the Biocompatibility of Multi-Anchored Glycoconjugate Functionalized Iron Oxide Nanoparticles in a Normal Human Colon Cell Line CCD-18Co

**DOI:** 10.3390/nano11102465

**Published:** 2021-09-22

**Authors:** Yash S. Raval, Anna Samstag, Cedric Taylor, Guohui Huang, Olin Thompson Mefford, Tzuen-Rong Jeremy Tzeng

**Affiliations:** 1 Department of Biological Sciences, Clemson University, Clemson, SC 29634, USA; yraval@g.clemson.edu (Y.S.R.); afogert@g.clemson.edu (A.S.); cedrict@g.clemson.edu (C.T.); ghuang@g.clemson.edu (G.H.); 2 Department of Materials Science and Engineering, Clemson University, Clemson, SC 29634, USA

**Keywords:** iron oxide nanoparticles, magnetic nanoparticles, colon cell toxicity, glycoconjugates, nanoparticle cellular uptake

## Abstract

We have previously demonstrated that iron oxide nanoparticles with dopamine-anchored heterobifunctional polyethylene oxide (PEO) polymer, namely PEO-IONPs, and bio-functionalized with sialic-acid specific glycoconjugate moiety (Neu5Ac(α2-3)Gal(β1-4)-Glcβ-sp), namely GM3-IONPs, can be effectively used as antibacterial agents against target *Escherichia coli*. In this study, we evaluated the biocompatibility of PEO-IONPs and GM3-IONPs in a normal human colon cell line CCD-18Co via measuring cell proliferation, membrane integrity, and intracellular adenosine triphosphate (ATP), glutathione GSH, dihydrorhodamine (DHR) 123, and caspase 3/7 levels. PEO-IONPs caused a significant decrease in cell viability at concentrations above 100 μg/mL whereas GM3-IONPs did not cause a significant decrease in cell viability even at the highest dose of 500 μg/mL. The ATP synthase activity of CCD-18Co was significantly diminished in the presence of PEO-IONPs but not GM3-IONPs. PEO-IONPs also compromised the membrane integrity of CCD-18Co. In contrast, cells exposed to GM3-IONPs showed significantly different cell morphology, but with no apparent membrane damage. The interaction of PEO-IONPs or GM3-IONPs with CCD-18Co resulted in a substantial decrease in the intracellular GSH levels in a time- and concentration-dependent manner. Conversely, levels of DHR-123 increased with IONP concentrations. Levels of caspase 3/7 proteins were found to be significantly elevated in cells exposed to PEO-IONPs. Based on the results, we assume GM3-IONPs to be biocompatible with CCD-18Co and could be further evaluated for selective killing of pathogens in vivo.

## 1. Introduction

There is a continuous rise in the literature that deals with the application of nanomaterials to treat various human diseases [1,2]. Currently, there exists a fundamental gap in translating the laboratory-based results of different nanoparticles to clinical scenarios. The primary factor that prevents their clinical application is inconsistent nanoparticle synthesis, which affects the physical and chemical properties of the nanoparticles as well as the nanoparticle stability in biological environments. In addition, there is a poor understanding of the interactions that occur between nanoparticles, biomolecules, and body fluids, and lastly the safety and biocompatibility of nanoparticles inside the human body [3,4].

This study focuses on the interactions between surface-functionalized iron oxide nanoparticles (IONPs) and various biological environments. IONPs were chosen for this study for their small size, unique magnetic properties, and high degree of biocompatibility. IONPs have garnered major attention in nanomaterials research due to their multitude of biomedical applications, which include but are not limited to targeted drug delivery, magnetic hyperthermia, magnetic resonance imaging, cell separation, cancer therapy, diagnostics, and pathogen detection [5,6,7]. During the typical synthesis procedure, via the thermal decomposition process, the resulting IONPs are generally found to be hydrophobic in nature and hence are colloidally unstable in a biological environment; thereby, they are not suitable in clinical applications [8,9]. Recent research efforts have largely concentrated on manipulating the surface chemical properties of IONPs to render them highly stable in biological-rich environments. Making such IONPs hydrophilic is of prime importance in order to achieve a chemically stable colloidal suspension of particles. For this purpose, surface coating of IONPs with biologically inert polymers is essential, as it will provide electrostatic and/or stearic repulsion and ‘stealth’ properties to IONPs in the presence of protein-rich biological environments. At the same time, the polymer-coated IONPs should be able to circulate in the body for a prolonged time duration until they reach their targeted location without triggering the body’s immune response [10,11].

Several studies have presented IONPs coated with appropriate polymer stabilizing agents, such as polyethylene oxide (PEO), also referred to as polyethylene glycol (PEG), to be highly biocompatible and biodegradable in vitro and in vivo [7,12,13,14]. PEO is generally regarded as a “stealth” polymer that is approved by the FDA. The major advantages of binding PEO onto nanoparticles for clinical applications are ease of manipulating their surface chemistry for widespread use in biomedical applications, long-term stability, amphiphilic nature, and solubility in water [8,15,16,17].

The main advantage of using IONPs for clinical applications is that, compared to other nanoparticles, IONPs can be metabolized and completely removed/excreted from the body through various systemic and cellular iron homeostasis pathways [18,19]. However, it should be carefully noted that the biocompatibility of IONPs is highly dependent on a multitude of factors like core size, surface chemistry of the IONPs, adsorbed proteins on the surface of IONPs, given dosage, biodistribution, and final localization of IONPs in the body [18,20,21,22]. Despite having excellent biocompatibility, numerous in vitro and in vivo studies have demonstrated differential toxicity of IONPs [19,22,23,24,25]. To date, the majority of the cell line toxicity studies of IONPs have been largely conducted in cancer cell line models [26,27,28,29,30]. However, using cancer cell lines does not always provide reliable nanotoxicity evaluation of tested nanomaterials since these cell lines may have been intentionally manipulated to make them immortal [31,32]. Toxicological studies carried out in normal or primary cells are warranted to fully comprehend and determine the possible toxicity mechanisms of IONPs before further testing is done in animal models.

PEO-coated IONPs further functionalized with carbohydrate molecules offer many advantages especially with regard to achieving a high affinity constant (K_a_) and increased binding enthalpy (ΔH) due to the presence of multivalent interactions [33]. On account of their high surface/volume ratio, functionalizing numerous carbohydrate groups onto the surface of PEO-coated IONPs is fairly easy and increases the biocompatibility of the entire nanoparticle system. These systems have been frequently used in various biomedical applications [33,34,35,36].

Few studies have described the use of nanoparticles functionalized with specific lectins and carbohydrates for targeted drug delivery to colon cells [37,38,39]. Of late, nanoparticles loaded with different drugs have been used as therapeutic agents for treating inflammatory bowel syndrome (IBS) [40,41]. Most of the in vitro studies that have been carried out to understand the cellular interaction of nanoparticles with intestinal cells used Caco-2 cell (human colorectal adenocarcinoma) monolayers [42,43,44,45]. However, using Caco-2 cells for such studies does not accurately reflect the physiological conditions of normal colon cells. Even though PEG/PEO polymer used for stabilizing IONPs has been reported to have excellent biocompatibility in numerous cell lines and animal studies, several research studies have deemed it to be toxic to cells [46,47,48,49,50]. Most of the in vitro cell line studies evaluating the toxicity of PEO-coated nanoparticles typically do not expose the cells above 100–200 μg/mL [18,51]. However, it is important to understand the biological response of cells in the presence of sub-lethal and lethal concentrations of nanoparticles. Depending on a variety of characteristics, PEO can show differential toxicity.

Previous research work has shown that IONPs synthesized with dopamine-anchored heterobifunctional PEO polymer (PEO-IONPs) and bio-functionalized with sialic-acid specific glycoconjugate moiety (Neu5Ac(α2-3)Gal(β1-4)-Glcβ-sp) (GM3-IONPs) can be effectively used as targeted antibacterial agents against enterotoxigenic *Escherichia coli*, which is usually associated with gastroenteritis and can also trigger post-infectious IBS [33,52]. This study will focus on evaluating the biocompatibility of both PEO-IONPs and GM3-IONPs in a range of concentrations on a normal human colon cell line CCD-18Co. The concentrations selected for this study were based on previous research that established the efficacy of the GM3-IONPs at inactivating *E. coli* [52]. Understanding the interactions occurring between different concentrations of IONPs and CCD-18Co cells can eventually help in determining the safe dosage levels of these IONPs to be effectively used as novel drug delivery agents for treating IBS and infections associated with it.

## 2. Materials and Methods

### 2.1. Synthesis of Iron Oxide Nanoparticles (IONPs)

IONPs were synthesized via thermal decomposition of an organometallic precursor in a high boiling point organic solvent following the procedures previously published by Raval et al. [33,53]. IONPs were imaged using a Hitachi 7600 TEM (Hitachi High-Technologies Corporation, Tokyo, Japan) (Supplementary Materials Figure S1A) prior to ligand exchange with PEO and the size distribution of the IONPs was calculated (Figure S1B).

### 2.2. Synthesis of Alkyne-PEO-PAA-Dopamine

PEO-PAA-Dopamine was synthesized following the procedure previously published by Stone et al. and then the solution was filtered, purified by dissolution in chloroform, and then precipitated in diethyl ether [54]. The final product was dried under vacuum and analyzed via proton nuclear magnetic resonance (HNMR) using a Jeol ECX-300 NMR (Peabody, MA, USA) and infrared spectroscopy (IR) on a Thermo-Nicolet Magna 550 FTIR spectrometer (Waltham, MA, USA) to confirm the presence of the catechol.

### 2.3. Ligand Exchange

IONPs as well as the PEO-PAA-dopamine were suspended separately in 5 mL of chloroform. The ligand exchange was run following the previously published work in Saville et al. and then the solutions were run through a gel permeation chromatography (GPC column) (Bio-Rad P polyacrylamide beads) to separate excess polymer from the water dispersible particles [55].

### 2.4. Click Chemistry

The click chemistry protocol was followed as seen in Raval et al. and the click reactions were left at room temperature for 12 h. They were then purified using size exclusion chromatography as previously reported [33,56].

### 2.5. Characterization of Functionalized IONPs

Dynamic light scattering (DLS) and zeta potential (Malvern Zetasizer Nano ZS, Malvern, UK) measurements were used to determine the nanoparticles hydrodynamic radius (Supplementary Materials Table S1). Additionally, Fourier transform infrared spectroscopy (FTIR) microscopy (Thermo-Nicolet Magna 550, Waltham, MA, USA) was performed to confirm the successful addition of ligand onto the surface of the particles via cycloaddition click-chemistry reaction. Inductively coupled plasma optical mass spectrometry (ICP-MS; Thermo-Scientific MS X Series II, Waltham, MA, USA) was performed to determine the concentration of iron [33].

### 2.6. Culturing of CCD-18Co Cells

CCD-18Co human normal colon cells (CRL-1459™) were procured from American Type Culture Collection (ATCC^®^) and routinely grown on 50 cm^2^ tissue-culture flasks in the presence of Dulbecco’s Minimum Essential Medium (DMEM; ATCC #30-2002). The DMEM was supplemented with 10% fetal bovine serum (FBS; Gibco -Thermo Fischer Scientific, Grand Island, NY, USA and 1% penicillin and streptomycin (Gibco—Thermo Fischer Scientific, Grand Island, NY, USA). CCD-18Co cultures were incubated at 37 °C in a humidified atmosphere of 5% CO_2_ and 95% air. Cells between passage generation of 12 and 25 were used to determine the biocompatibility of IONPs. CCD-18Co cells were seeded into 96-well plates at a density of 1.5 × 10^4^ cells/well and allowed to adhere for 24 h. The cells were then treated with DMEM supplemented with PEO-IONPs or GM3-IONPs at final concentrations of 10, 50, 100, 250, or 500 μg/mL and incubated for 24 or 48 h unless otherwise indicated.

### 2.7. Cytotoxicity of IONPs to CCD-18Co Cells

The potential cytotoxicity of PEO-IONPs and GM3-IONPs towards CCD-18Co cells was determined by performing the MTS assay (CellTiter 96^®^ Aqueous One Solution Cell Proliferation Assay, Promega, Madison, WI, USA). The plate was read at 490 nm in a microplate reader (Synergy Hybrid H1, Biotek^®^).

### 2.8. Intracellular Adenosine Triphosphate (ATP) Levels of CCD-18Co Cells in the Presence of IONPs

Intracellular ATP levels of CCD-18Co cells were measured in the presence of PEO-IONPs or GM3-MNPs using the CellTiter-Glo^®^ 2.0 assay (Promega) to determine if their presence interrupted/inhibited ATP synthesis in the cells. At the end of this assay, the plate was read in a micro-plate reader with luminescence capability (Synergy Hybrid H1, Biotek^®^) and the obtained results were expressed in relative luminescent units (RLUs) [57,58].

### 2.9. Cell Membrane Integrity of CCD-18Co Cells in the Presence of IONPs

A live/dead^®^ viability assay (Invitrogen, Eugene, OR, USA) was performed to determine the extent of cell membrane damage of CCD-18Co cells in the presence of DMEM supplemented with PEO-IONPs and GM3-IONPs at a final concentration of 500 μg/mL. The stained cells were observed under a fluorescent microscope (Motic AE 30, Schertz, TX, USA) with appropriate fluorescent filter cubes at 100X and 200X magnification. Later, the images obtained under different fluorescent filters were merged in ImageJ software (NIH, Bethesda, MD USA) [23,59].

### 2.10. Intracellular Glutathione (GSH) Levels of CCD-18Co Cells in the Presence of IONPs

Intracellular GSH levels were measured by utilizing the GSH-Glo™ Glutathione assay kit (Promega). At the end of this assay, the plate was read in a micro-plate reader (Synergy Hybrid H1, Biotek^®^, Winooski, VT, USA) and the luminescence intensity of each well obtained was expressed in relative luminescent units (RLUs) [45,60].

### 2.11. Intracellular Detection of ROS Using Dihydrorhodamine-123 (DHR-123) in CCD-18Co Cells in the Presence of IONPs

Intracellular levels of ROS were indirectly measured through the oxidation of dihydrorhodamine-123 (DHR-123, Invitrogen™, Eugene, OR, USA) CCD-18Co cells treated with IONPs had the media removed, were washed with sterile PBS, and then covered with PBS containing 5 μM DHR-123. The plate was incubated in the incubator for 60 min and then read on a micro-plate reader (Synergy Hybrid H1, Biotek^®^, Winooski, VT, USA) at an excitation of 500 nm and emission of 536 nm.

### 2.12. Intracellular Caspase 3/7 Levels of CCD-18Co Cells in the Presence of IONPs

Intracellular caspase 3/7 protein levels were determined through the Caspase-Glo^®^ 3/7 assay kit (Promega). The plate was read in a micro-plate reader (Synergy Hybrid H1, Biotek^®^) and luminescence intensity of each well obtained was expressed in relative luminescent units (RLUs) [61,62].

### 2.13. Inductively Coupled Plasma Optical Mass Spectrometry (ICP-MS)

ICP-MS (Thermo Fisher Scientific MS X Series II, Waltham, MA, USA) was run using only ^56^Fe for analysis with ^45^Sc as an internal standard. Cell samples were incinerated at 700 °C for 6 h to burn off all organic material. Samples were then digested with Aqua regia (a mixture of nitric acid and hydrochloric acid at an optimal molar ratio of 1:3) to remove any residual organic/char. The remaining inorganic was digested with 70% nitric acid. The nitric acid was boiled off and the resulting nitrate salts were dissolved in a known volume of 2% nitric acid solution. Unknown samples were run against a known set of standards between 0.01 and 100 ppm iron to calculate the concentration.

### 2.14. Transmission Electron Microscopy (TEM) Images of IONPs Uptake

IONPs uptake by the cells was observed using TEM. These were analyzed for possible cell damage at high concentrations of nanoparticle treatment. CCD-18Co cells were seeded into T-75 flasks (Corning, NY, USA). Following 24-h incubation, PEO-IONPs or GM3-IONPs were added at a concentration of 100 μg/mL. After a 24- or 48-h incubation period, the cells were fixed and imbedded in London Resin (LR) white embedding medium following the protocol seen in Fellows et al. [63]. Ultra-thin sections (70–90 nm) were cut using a microtome and mounted on formvar coated copper grids. A Transmission Electron Microscope Hitachi H7600 (Hitachi High-Technologies Corporation, Tokyo, Japan) was used for imaging.

### 2.15. Statistical Analysis

The statistical analysis was performed using GraphPad Prism software (V 7.0, San Diego, CA, USA). The experiments were done in triplicates and data are expressed as Mean ± SD. Statistically significant differences between the groups were evaluated by performing ANOVA. Post hoc group comparisons were calculated through Tukey’s multiple comparisons test. Results showing *p*-values of ≤0.05, <0.01, and <0.001 were considered to be statistically significant.

## 3. Results and Discussion

### 3.1. IONPs Stability in Cell Culture Medium

The presence of biological medium has an important effect on the overall size diameter and surface charge of the synthesized IONPs. The presence of functional groups on the surface of IONPs also determines the extent to which it interacts with salts and proteins. The synthesized PEO-IONPs and GM3-IONPs were characterized by TEM (Figure S1). Further details on the click chemistry functionalization process can be found in Figure S2. Further material characterization results of the IONPs are also given in Figures S3–S5. DLS studies were conducted both on PEO-IONPs and GM3-IONPs over a period of 3 days to evaluate their overall stability in cell culture medium DMEM in the absence/presence of 10% FBS. As seen from Table 1, the particle size of PEO-IONPs (in water) instantly increased from 78.8 nm to 173.03 nm within 5 min of incubation in the presence of DMEM. After 72 h, the particle size still remained ~170 nm. The presence of FBS in DMEM did not significantly change the overall diameter of PEO-IONPs during the entire experimental period. However, we did notice a slight reduction in the size of PEO-IONPs in the presence of DMEM+FBS. In contrast, GM3-IONPs (in water) non-significantly increased their size from 88.8 nm to 104 nm when mixed and incubated for 5 min in the presence of DMEM. In addition, the overall size of GM3-IONPs did not change drastically over the 72-h incubation time-period in the presence of DMEM. After mixing GM3-IONPs with media containing DMEM+FBS for 5 min, there was no statistically significant change in the diameter. However, after 24 h, the size increased to ~106 nm in the presence of DMEM+FBS. Additional DLS intensity graphs are also given in Figures S6 and S7. Several studies reported the formation of a ‘nanoparticle corona’ layer on the surface of IONPs when incubated in the presence of cell culture medium containing high salt and protein concentrations [18,64,65]. This nanoparticle-corona formation can be mainly attributed to either electrostatic interactions, van der Waals, covalent interactions, hydrophobic interactions, or steric interactions occurring between the different amino acids, salts, and other biomolecules present in the cell culture media. It is due to this nanoparticle-corona formation that the overall size of the nanoparticles is increased. Moreover, an extended period of nanoparticle-corona formation can also affect the colloidal stability of the nanoparticle system in the biological media and eventually the nanoparticles tend to lose their colloidal stability and form aggregates due to nanoparticle-corona formation.

The presence of different types of chemical functional groups found on polymers and the length of the polymer itself plays an important role as to how and what kind of proteins interact with the nanoparticle surface and become adsorbed on it [66,67]. The results portray a rapid increase in the size diameter of PEO-IONPs within 5 min of incubation with DMEM. After 5 min, the size remained the same for the next 72 h. Such a phenomenon can be credited to formation of a thick layer of ‘hard nanoparticle-corona’, which essentially represents an irreversible change in the amounts of biomolecules, ionic salts, and proteins that are getting adsorbed/released over a period of time on the surface of nanoparticles [68,69]. Albumin and globulins are the most dominant serum proteins found in any cell culture medium [66]. Major ionic salts that are present in cell culture media include sodium (Na^+^), potassium (K^+^), chloride (Cl^−^), and bicarbonate (HCO_3_^−^). The PEO-PAA polymer that we used as multi-anchored stabilizing agent for PEO-IONPs has a lot of reactive alkyne groups on its surface that are free and can potentially interact with the above-mentioned serum proteins and ionic salts present in DMEM. A study done by Ekkebus et al. showed that terminal alkyne groups could selectively react with cysteine amino acid via thiol-alkyne side chain reaction [70]. The size increase of PEO-IONPs when mixed with DMEM could be due to the chemical interactions taking place between the free alkyne groups present on our polymer chains with a thiol end-group containing amino acids like cysteine and cystine. Moreover, several coenzymes and cofactors present in DMEM contain thiol groups, which can also interact with alkyne. The above-mentioned chemical reactions taking place between alkyne- and thiol-rich compounds might be one of the reasons for the formation of the nanoparticle-corona around PEO-IONPs in DMEM and thereby increasing its overall diameter size by more than two-fold. In the case of GM3-IONPs, the PEO-PAA polymer with the alkyne group underwent a ‘click reaction’ to covalently attach the GM3 molecule through an alkyne-azide linkage. So, GM3-IONPs would have a relatively less amount of free alkyne groups that can interact with amino acids and ionic salts of DMEM, and hence less amount of biomolecules would get adsorbed on its surface and form a thin layer of nanoparticle-corona, which would eventually increase the overall size of GM3-IONPs. Furthermore, it is worthwhile to note that the presence of a multi-anchored DOPA group is also responsible for maintaining the colloidal stability of IONPs in a biological environment via intact stearic interactions. The work of Stone et al. suggested that multi-anchored dopamine groups present on IONPs made them colloidally stable in FBS medium compared to mono-anchored groups that lost their stability by forming large aggregates with a size diameter of >500 nm [54].

### 3.2. MTS Assay

**Figure 1:**
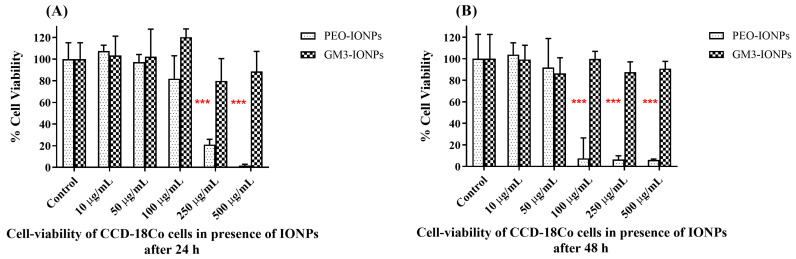
Cell viability cytotoxicity MTS assay: (**A**) CCD-18Co cells exposed to varying concentrations of PEO-IONPs for 24 h; (**B**) CCD-18Co cells exposed to varying concentrations of GM3-IONPs for 48 h. Data is expressed as Mean ± SD (*n* = 3); Statistical analysis—Analysis of Variance (ANOVA); *** *p*-value < 0.001.

The presence of nanomaterials in cell lines is one of the many factors responsible for inducing toxicity in them. Different nanoparticles, when presented with direct contact to cells, can elicit cytotoxic responses inside mitochondria. One of the many in vitro assays, which determine the damage done to mitochondria in presence of nanoparticles, is to quantify and measure the reductase/dehydrogenase enzyme’s activity inside the living mitochondria [20,71]. The MTS assay is one of the frequently employed cytotoxicity assays, which measures the amount of tetrazolium salt that is bio-reduced to formazan product by viable cells. This amount can then be detected colorimeterically and formazan produced is directly proportional to the number of living cells. The cytotoxicity of PEO-IONPs and GM3-IONPs to CCD-18Co cells was measured by the CellTiter 96^®^ Aqueous One Solution Cell Proliferation Assay [33]. Increasing concentrations of both IONPs were added and the cells were incubated for 24 and 48 h. As seen from Figure 1A, PEO-IONPs were found to be highly cytotoxic to CCD-18Co cells above the 100 μg/mL concentration after 24 h of exposure in a dose-dependent manner. Further, <5% cell viability was observed in the cells exposed to the 500 μg/mL concentration of PEO-MNPs at the end of 24 h. The GM3-IONPs did not show any significant cytotoxicity at all concentrations. In comparison to the 24-h time-period, PEO-IONPs showed significant cytotoxicity to the cells after 48 h of exposure even at the 100 μg/mL concentration (Figure 1B). Above a concentration of 100 μg/mL, <5% cell viability of CCD-18Co cells was seen. To our surprise, cells exposed to GM3-IONPs for 48 h did not show any significant cytotoxicity. Even the maximum concentration of 500 μg/mL of GM3-IONPs showed a >90% viability rate. PEO polymers attached to nanoparticle surfaces are generally found to be biocompatible both in in vitro and in vivo settings [15,16,72]. However, there have been few reports of toxicity of PEO-coated nanoparticles. In a recent study conducted by Escamilla-Rivera et al., IONPs coated with PEG were found to result in 50% cell viability at a 100 μg/mL concentration after 48 h of exposure to THP-1 macrophages [73]. Additionally, the presence of the PAA group on the polymer has been described to have significant toxicity in animal models [47,74,75]. It should be noted that the cell viability rate also depends on the length of PEO tails present on the surface of IONPs as reported by Hafeli et al. [76]. Their study showed that increasing the tail length/size of PEO (from 0.75 kDa to 15 kDa), the cell viability rate of various human cell lines also increased. Similar to the results seen here, the presence of carbohydrate/glycoconjugate molecules on IONPs have been reported to have no significant cytotoxicity to different cell lines [77,78,79].

### 3.3. Intracellular Adenosine Triphosphate Assay

**Figure 2:**
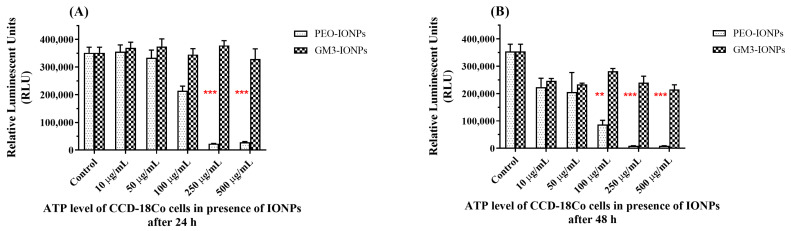
Intracellular ATP levels of CCD-18Co cells in the presence of IONPs using CellTiter Glo 2.0 assay. (**A**) ATP levels of cells exposed to PEO-IONPs at increasing concentrations for 24 h; (**B**) ATP levels of cells exposed to GM3-IONPs at increasing concentrations for 48 h. Data is expressed as Mean ± SD (*n* = 3); Statistical analysis—Analysis of Variance (ANOVA); ** *p*-value < 0.01, and *** *p*-value < 0.001.

The amount of ATP level present in any cell determines its metabolic state. In the presence of toxic materials/chemicals, the metabolic state of the cell will change, and the intracellular ATP levels can drop if there is any significant cytotoxicity to the cells. Higher levels of intracellular ATP commonly indicate that the cell is metabolically active, and their level directly correlates to the actual number of living cells. Research studies have reported a significant decrease in intracellular ATP levels of cells in the presence of different types of nanoparticles [80,81,82]. To further understand the inherent cytotoxic mechanisms of IONPs on the inner cell membrane biochemical cycles taking place inside mitochondria, we measured the intracellular ATP levels of CCD-18Co cells utilizing a luminescent assay. The cells were incubated with different concentrations of PEO-IONPs or GM3-IONPs for 24 and 48 h. As seen from Figure 2A,B, the ATP levels of the cells started to decrease substantially when exposed to PEO-IONPs above a 100 μg/mL concentration. More than a 90% reduction in the ATP levels was observed for 250 μg/mL and 500 μg/mL concentrations of PEO-IONPs. In comparison, cells exposed to GM3-IONPs for 24 h did not show any significant decrease in ATP levels at all concentrations. When the exposure time was increased to 48 h, cells in the presence of PEO-IONPs showed a rapid decline in intracellular ATP levels beginning from the 100 μg/mL concentration (Figure 2B). At concentrations above 100 μg/mL, a significant reduction (>95%) in ATP levels was seen in the presence of PEO-IONPs. Several reports have suggested that the kind of polymer coating and size of IONPs could play an important role in maintaining ATP levels inside the cells. In one such study, IONPs coated with different polymers like DEAE, chitosan, and PEI exhibited variation in its cytotoxic response to a human brain microvascular endothelial cell line, with PEI-IONPs showing maximum cytotoxicity [83]. In another study, IONPs functionalized with starch were incubated with a murine macrophage cell line for 48 h and the authors observed a drastic decrease in ATP levels of the cells [84]. However, the presence of GM3-IONPs to the CCD-18Co cells only resulted in a negligible decrease in ATP levels at concentrations above 250 μg/mL, thereby, showing that GM3-IONPs do not cause any detrimental toxic effects on the overall functioning of ATP synthesis mechanisms inside mitochondria in CCD-18Co cells. These results show that PEO-IONPs can possibly interfere with the ATP synthesis pathways inside the mitochondrial membrane, which can cause a reduction in proton motive force and membrane depolarization.

### 3.4. Cell Membrane Integrity Assay

**Figure 3:**
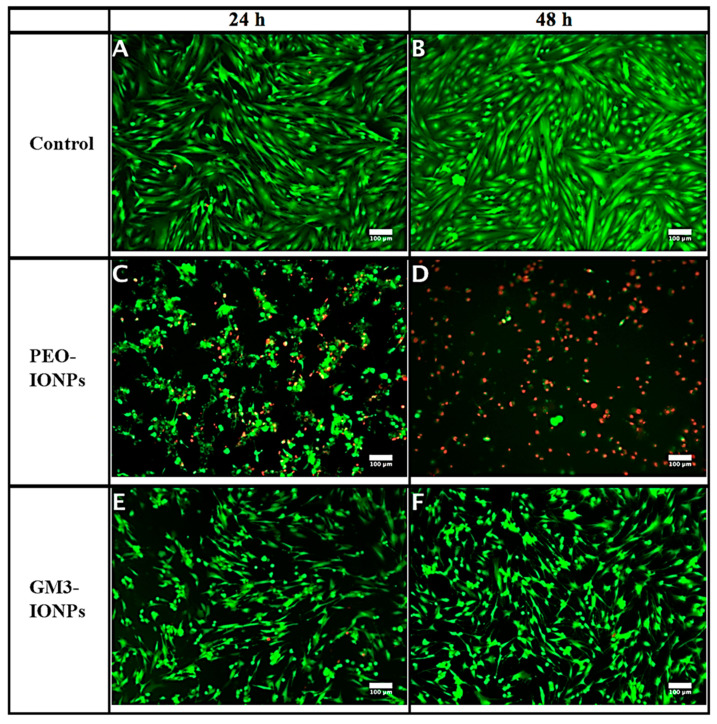
Live/dead staining assay using calcein AM and ethidium homodimer-1 dyes. CCD-18Co cells were incubated in presence of PEO-IONPs and GM3-IONPs for 24 and 48 h (Concentration—500 μg/mL). Live cells appear green in color and dead cells appear red in color. All the images were merged together for both green and red channel filters of the microscope. (**A**,**B**)—control cells (no IONPs); (**C**,**D**)—cells exposed to PEO-IONPs; (**E**,**F**)—cells exposed to GM3-IONPs. Magnification: 100×; Scale bar—100 μm.

Next, we evaluated the toxicity of IONPs to CCD-18Co cells by performing the membrane integrity assay. It is one of the commonly employed in vitro assays that determine the extent of membrane damage of mammalian cells when exposed to various nanomaterials [51,71]. Most of these assays utilize a mixture of fluorescent dyes that can interact with specific enzymes present inside the cells depending on their ability to enter the cell. For this study, we exposed the cells to the highest concentration of IONPs (500 μg/mL) based on the preliminary cytotoxicity results that we observed. After the predetermined incubation time-period, the live/dead viability assay was done in the presence of two fluorescent dyes, calcein AM and ethidium homodimer-1. Calcein AM is a cell-permeable dye, which is non-fluorescent to begin with. Once inside the living cell, various intracellular esterases break down this dye and it is retained inside the cells that have intact membranes and can now emit intensely green fluorescence. Contrary, ethidium homodimer-1 dye cannot enter live cells that has intact membranes. Those cells whose cell membrane integrity has been compromised will take it up and once inside these damaged cells, it can brightly emit red fluorescence. As seen from Figure 3C, CCD-18Co cells exposed to PEO-IONPs after 24 h showed a distinct change in their morphology and structure compared to control cells, which showed perfect slender and elongated fibrils (Figure 3A). The cells were found to be clumped together in small irregular round/oval shapes. There was partial membrane damage to the cells as evident from the limited red fluorescence. After 48-h exposure, the majority of the cells exposed to PEO-IONPs suffered extensive membrane damage (as seen by intense red fluorescence) and the cells also shrunk in size (Figure 3D). We also observed extensive cell detachment in CCD-18Co cells in the presence of PEO-IONPs. Such cell features are indicative of necrosis/ apoptosis. In comparison, cells incubated with 500 μg/mL of GM3-IONPs for 24 and 48 h did not show any visible cell membrane damage (Figure 3E,F). More than 90% viability was seen in these cells. However, there was a small change in the overall size and arrangement of the cells. Compared to the control group, cells exposed to GM3-IONPs had smaller fibrils and their overall size dimensions were slightly reduced. These cells also grew at a further distance from each other and there was hardly any overlapping of cell fibrils with each other indicating cell retraction. One possible explanation for such morphological change in cells in the presence of IONPs might have to do with the maintenance of cytoskeleton structures that includes actin and tubulin filaments. Both these structures are essential for proper growth and maintenance of cells as they directly take part in cell–cell communication, transport of nutrients, and other vital organelles. Research studies have reported that IONPs functionalized with dextran, citric acid, and PEG can effectively disrupt the overall cytoskeleton arrangement in different cell lines through destruction of actin and microtubules through cell uptake [59,85,86]. Additionally, at high concentrations of IONPs (500 μg/mL and 1000 μg/mL), the overall length and diameter of murine neural progenitor cells and primary human blood outgrowth endothelial cells were found to be condensed and these cells also showed retraction properties during their growth cycle, which is similar to what we observed in CCD-18Co cells exposed to GM3-IONPs [87]. It also reduced the expression of focal adhesion kinase (FAK) protein, which is suggested to have damaging repercussions on kinase signaling pathways that maintain the cytoskeleton structures. Such disruptions in cytoskeleton pathways can activate pro-apoptosis signaling pathways in the cells, which can lead to cell death. Thus, the results of our live/dead staining assay suggest that PEO-IONPs can possibly interfere and destroy cell cytoskeleton structures that can stall the regular cell cycle and cause cell death whereas, the presence of the GM3 molecule on IONPs can potentially prevent such drastic cytoskeleton toxicity to CCD-18Co cells.

### 3.5. Intracellular Glutathione Assay

**Figure 4:**
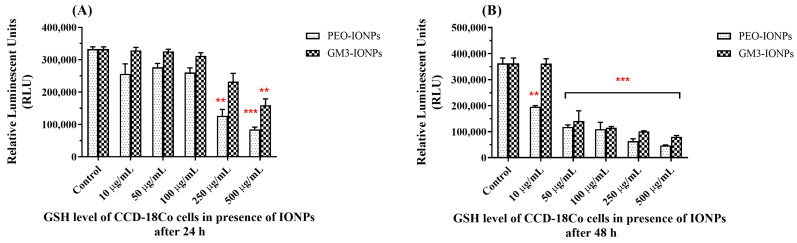
Intracellular glutathione (GSH) levels of CCD-18Co cells in the presence of IONPs using the GSH-Glo assay. (**A**) GSH levels of cells exposed to PEO-IONPs at increasing concentrations for 24 h; (**B**) GSH levels of cells exposed to GM3-IONPs at increasing concentrations for 48 h. Data is expressed as Mean ± SD (*n* = 3); Statistical analysis—Analysis of Variance (ANOVA); ** *p*-value < 0.01, and *** *p*-value < 0.001.

It is known that the amount of reactive oxygen species in the cellular environment gives an indication of the oxidative stress levels. Glutathione (GSH) is an important and powerful antioxidant present in the mammalian cell and normally exists in a reduced state. However, when the cell is experiencing oxidative stress due to the presence of reactive oxygen species (such as singlet oxygen, hydroxyl, and superoxide radicals), reactive nitrogen species, and metal ions, GSH is converted into its oxidized dimeric form GSSH. This can serve as an indicator of cellular oxidative stress that can ultimately lead to cell death or apoptosis [88,89]. There have been numerous reports of increased oxidative stress in cells exposed/treated with different kinds of nanomaterials, especially IONPs [90,91,92]. In order to evaluate whether CCD-18Co cells underwent cellular oxidative stress in the presence of IONPs, we carried out the GSH-Glo™ assay to check for any changes in the overall GSH levels. At the end of 24 h of exposure to PEO-IONPs, the GSH levels in the cells decreased at particle concentrations of 250 μg/mL and 500 μg/mL (Figure 4A). In the case of GM3-IONPs, cells lowered their overall GSH counts only at the highest concentration of 500 μg/mL. Compared to 24 h, cells exposed to 48 h of PEO-IONPs and GM3-IONPs exhibited a concentration-dependent decrease in GSH levels starting from the 50 μg/mL concentration (Figure 4B). The overall reduction in GSH levels is supported in the literature as numerous works have examined the effect of IONPs in disturbing the overall mechanisms of antioxidant pathways in cells [93,94,95]. The most common in vitro and in vivo toxicity of IONPs develop due to production of reactive oxygen species (ROS), which include singlet oxygen, hydrogen peroxide, hydroxyl radicals, and superoxide anions [90,96]. IONPs are likely to be taken up by the cells via different endocytic pathways depending on their size and surface chemistry [97]. Once inside the cells, IONPs are typically degraded in the lysosomes into ferrous (Fe^2+^) ions due to their low pH environment. These Fe^2+^ ions potentially enter the mitochondrial membrane system through membrane depolarization and interact with different enzymes of the electron transport system [19,91]. High levels of ROS species can deteriorate the cellular levels of GSH, thereby causing oxidative stress. A study conducted by Watanabe et al. reported that exposing human alveolar epithelial cells to IONPs caused DNA damage, increased ROS production, and reduced GSH levels even at low concentrations of 10 μg/mL, which is similar to what we observed [98]. Similarly, a significant increase in ROS levels and simultaneous reduction in GSH levels were observed in human breast cancer cells when exposed to IONPs. Here too, the authors described these effects to be time and concentration dependent [99]. Based on these results, we can aptly deduce that both types of IONPs systems used in our experiments are responsible for generating increased ROS levels along with reduced intracellular GSH levels in CCD-18Co cells. Further studies need to be conducted to determine the precise levels of several intracellular ROS that are getting boosted due to the presence of IONPs to fully elucidate the role of ROS in causing cellular toxicity in the presence of IONPs.

### 3.6. Intracellular Caspase 3/7 Assay

**Figure 5:**
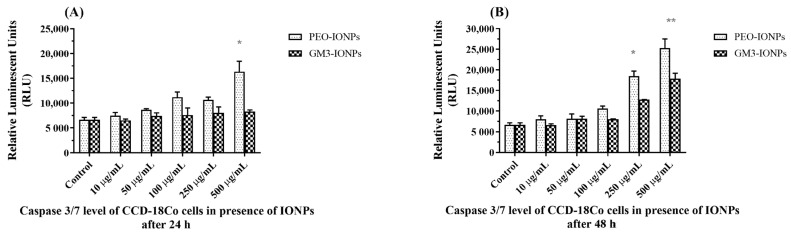
Intracellular caspase3/7 activity levels in CCD-18Co cells exposed to IONPs using the Caspase-Glo 3/7 assay. (**A**) Caspase3/7 levels of cells exposed to PEO-IONPs at increasing concentrations for 24 h; (**B**) Caspase3/7 levels of cells exposed to GM3-IONPs at increasing concentrations for 48 h. Data is expressed as Mean ± SD (*n* = 3); Statistical analysis—Analysis of Variance (ANOVA); * *p*-value < 0.05, ** *p*-value < 0.01.

The presence of nanomaterials in biological systems induces apoptosis in cells by activating various cell death signaling pathways [25,100]. To determine whether CCD-18Co cells are showing any apoptotic activity in the presence of IONPs, we quantitatively measured the levels of caspase3 and caspase7 proteins [101,102]. Upon detecting a major change in the normal biochemical processes occurring inside the mitochondria, different signaling pathways are activated, which can trigger the activation of caspase proteins. One of the last proteins of the caspase family to get activated before the cell inadvertently goes into the cell death stage is caspase3 [103]. So, we performed the Caspase-Glo^®^ 3/7 assay on CCD-18Co cells exposed to IONPs to determine if their presence activated the caspase signaling pathways leading to apoptosis. After incubating the cells with PEO-IONPs, there was no significant change in the levels of caspase3/7 proteins up to the 250 μg/mL concentrations (Figure 5A). However, cells exposed to 500 μg/mL PEO-IONPs showed a substantial increase in activity of caspase3/7 proteins, which suggests that the cells might be undergoing apoptosis. In comparison, cells incubated with GM3-IONPs for 24 h maintained similar caspase3/7 levels at all concentrations. When the exposure time of IONPs to cells was increased to 48 h, cells incubated with both the 250 μg/mL and 500 μg/mL concentrations of PEO-IONPs showed a notable rise in caspase3/7 levels (Figure 5B). This shows that even at a concentration of 250 μg/mL of PEO-IONPs, the cells may be experiencing apoptosis. When GM3-IONPs were incubated with cells for 48 h, there was no significant change in caspase levels until the concentration reached 500 μg/mL. These results suggest that both PEO-IONPs and GM3-IONPs are able to activate caspase signaling events in CCD-18Co cells only when presented with the highest concentrations of IONPs. However, it seems that the exposure time does play a critical role in induction of apoptosis as the overall caspase levels were elevated at 48 h compared to 24 h. Similar results were obtained in several research articles, which showed a time-dependent increase in caspase protein levels in presence of IONPs [62].

In terms of the concentration-dependent rise in caspase levels, the results in this experiment differ from what has been reported earlier. In one of the studies done by Yin et al., the caspase3 levels in rat cerebellum cells showed a dose-dependent increasing trend when exposed to silver nanoparticles [104]. In another study, the size and surface functionalization of polystyrene latex nanoparticles played a significant part in initiating caspase-dependent apoptotic pathways in human alveolar epithelial cells [105]. They noticed that cells exposed to 100 nm amine-coated nanoparticles had significantly higher levels of caspase proteins compared to those exposed to 50 nm-sized nanoparticles and also to carboxyl-coated nanoparticles. Hence, based on our results of the caspase3/7 assay, it seems that the surface chemistry and exposure time are important parameters to consider when using IONPs for therapeutic applications. The levels of other apoptotic proteins need to be determined in order to completely understand if the cellular toxicity mechanism in the presence of IONPs is indeed due to the activation of apoptosis signaling pathways.

### 3.7. Inductively Coupled Plasma Optical Mass Spectrometry

**Figure 6:**
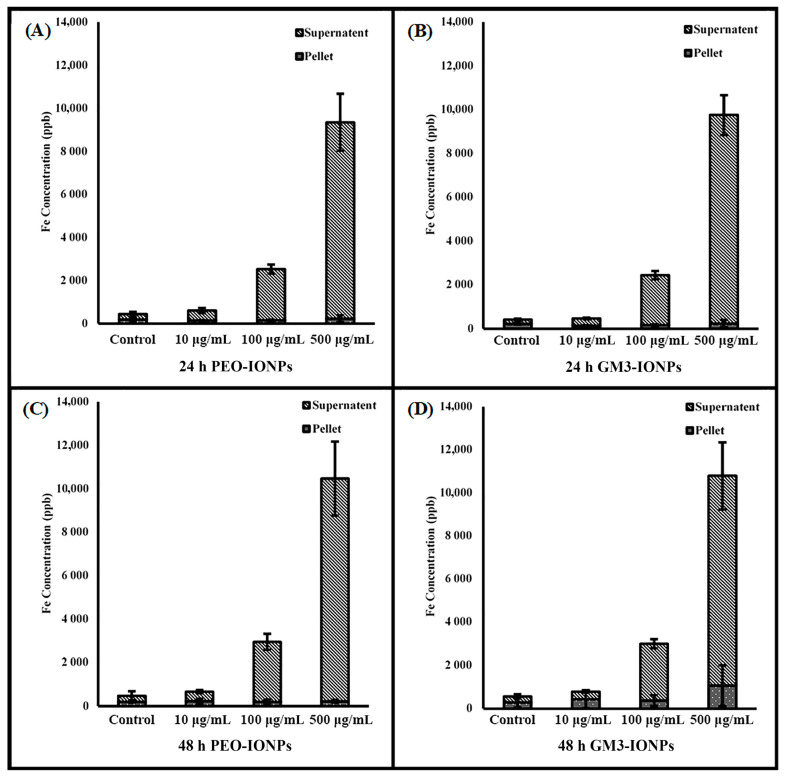
Comparing supernatant to cell pellet in CCD-18Co cell uptake of PEO- and GM3-IONPs measured in Fe concentration using ICP-MS (**A**) Fe levels of cell pellet/supernatant exposed to PEO-IONPs at increasing concentrations for 24 h; (**B**) Fe levels of cell pellet/supernatant exposed to GM3-IONPs at increasing concentrations for 24 h; (**C**) Fe levels of cell pellet/supernatant exposed to PEO-IONPs at increasing concentrations for 48 h; (**D**) Fe levels of cell pellet/supernatant exposed to GM3-IONPs at increasing concentrations for 48 h. Data is expressed as Mean ± SD (*n* = 3).

**Figure 7:**
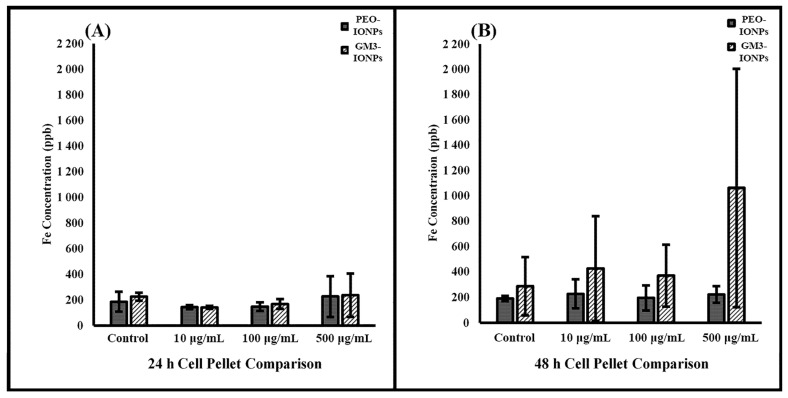
Comparing cell pellet in CCD-18Co cell uptake of PEO- and GM3-IONPs measured in Fe concentration using ICP-MS (**A**) Fe levels of cell pellet exposed to PEO-IONPs at increasing concentrations for 24 versus 48 h; (**B**) Fe levels of cell pellet exposed to GM3-IONPs at increasing concentrations for 24 versus 48 h. Data is expressed as Mean ± SD (*n* = 3).

To further evaluate the potential toxicity of IONPs to CCD-18Co cells, an ICP-MS experiment was performed. ICP-MS combines a high-temperature ICP source with a mass spectrometer and the ICP source converts the atoms of the elements in the sample to ions. These ions are then separated and detected by the mass spectrometer, allowing it to detect elements at low concentrations. Concentration effects and incubation times are two variables that were analyzed in the interest of isolating the different roles of PEO- and GM3-IONPs particles on cell uptake. Research has looked into optimizing the uptake of iron oxide nanoparticles into cells for a variety of biomedical objectives [106,107,108]. After incubating the cells for 24 or 48 h with PEO- or GM3-IONPs at different concentrations, the results in Figure 6 show that the majority of the IONPs remained in the supernatant. Care was taken to wash the cell pellets multiple times to ensure only nanoparticles attached or taken up into the cells would remain with the cell pellet. The remaining nanoparticles were washed off and combined with the supernatant category. Figure 7 analyzes the pellets’ uptake of PEO- and GM3-IONPs. It can be seen that there was a steady amount of iron uptake throughout the 24- and 48-h incubation times for the different concentrations of PEO-IONPs. However, there is an increase in iron for the GM3-IONPs 48-h category. A plausible explanation of this result is due to the number of cells in the cell pellets. The fluorescent images, represented in Figure 3D,F, indicate that the PEO-IONPs-treated cells do not remain attached to the cell culture flasks after being put under stress. This can be observed in the rounding/shrinking of their overall shape. Comparing the stress the cells exhibit when treated with the PEO-IONPs versus the GM3-IONPs, it is possible that many of the detached apoptotic cells in the PEO-IONPs category might have been included in the supernatant rather than the cell pellet. In order to investigate this, cell counts were taken of the supernatant removed from cells treated with PEO- or GM3-IONPs. No intact cells were observed in either group. This observation fits with the cellular toxicity that PEO-IONPs exhibit towards CCD-18Co cells.

Although the ICP cell uptake data showed an increase in the cell pellet iron concentration for the GM3-IONPs, it is possible that this increase was due to the number of cells present in the cell pellets. With the increased stress of the PEO-IONPs treatment on the CCD-18Co cells, it is likely that the cells became detached, resulting in an overall lower number of cells in the cell pellet when compared to the GM3-IONPs treatment. This could lead to an artificially inflated iron concentration in the pellet of the GM3-IONPs-treated group when compared to the pellet of the PEO-IONPs group. In order to evaluate this, we attempted to count the detached cells in the supernatant of cells treated with IONPs. However, no whole cells were observed, leading to the conclusion that due to the incubation time and stress, it caused the cells in the supernatant to lose membrane integrity and allowing intracellular PEO-IONPs to be released into the supernatant. The elevation of iron observed in the GM3-IONPs pellet could potentially be attributed to the limited uptake of GM3-IONPs observed.

### 3.8. Transmission Electron Microscopy

**Figure 8:**
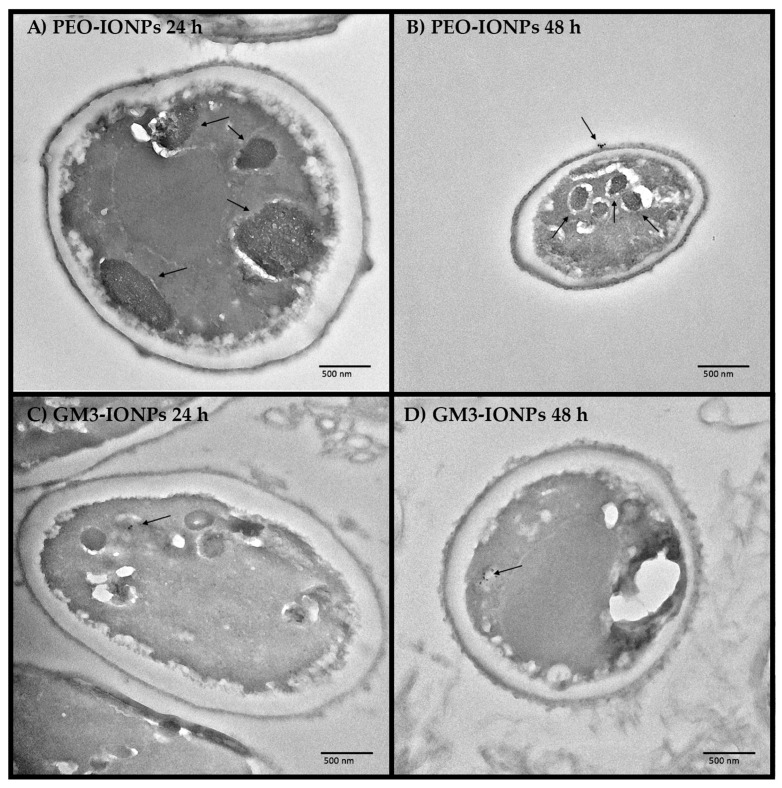
Transmission electron microscopy (TEM) Images of CCD-18Co cells incubated in the presence of PEO-IONPs and GM3-IONPs for 24 and 48 h (Concentration—100 μg/mL). (**A**) CCD-18Co cells exposed to PEO-IONPs and incubated for 24 h; (**B**) CCD-18Co cells exposed to PEO-IONPs and incubated for 48 h; (**C**) CCD-18Co cells exposed to GM3-IONPs and incubated for 24 h; (**D**) CCD-18Co cells exposed to GM3-IONPs and incubated for 48 h; Scale bar—500 nm. The arrows indicate the uptake of nanoparticles.

TEM images were captured to visualize the cellular uptake of PEO- and GM3-IONPs within CCD-18Co cells. After incubating the cells for 24 or 48 h with 100 μg/mL PEO- or GM3-IONPs, the cell pellets were thoroughly washed to remove unattached nanoparticles. These pellets were fixed in resin and sliced on a microtome for TEM imaging. The TEM images shown in Figure 8 compare the difference of PEO-IONPs and GM3-IONPs uptake into the cells. It should be noted that the organelles inside the PEO-IONPs-treated cells, seen in Figure 8A,B, have a much darker shade and larger size when compared to the GM3-IONPs group, potentially indicating nanoparticle uptake into these organelles. Additionally, in Figure 8B, there is indication of endocytosis with a group of nanoparticles attached and entering the CCD-18Co cell membrane. In the GM3-IONPs test group, seen in Figure 8C,D, there is a small amount of uptake visible within the cells as individual nanoparticles are distinguished, but no large grouping of nanoparticles is visible, indicating the lack of substantial GM3-IONPs uptake by the cells. One possible reason to explain this is the difference in the uptake of nanoparticles can be attributed to the difference in the overall size and surface chemistry of the IONPs. PEO-IONPs were found to be larger in size diameter compared to GM3-IONPs. Moreover, they have more free functional groups of their surface compared to GM3-IONPs. Several studies have reported that both nanoparticle size and its surface chemistry dictates how many particles can enter into the cell [43,47,49,60,61,69,97,107].

Additionally, the TEM images of cell uptake showed that some uptake does occur with both the PEO-IONPs and GM3-IONPs when incubated with 100 μg/mL samples for 24 or 48 h. However, the images suggest that more IONP uptake was seen in the PEO-IONPs test groups, indicating a potential correlation between PEO-IONPs uptake and cellular toxicity (Figure 8).

## 4. Conclusions

We successfully synthesized multi-anchored glycoconjugate functionalized GM3-IONPs based a ‘click-chemistry’ platform that are stabilized with heterobifunctional PEO-PAA polymer having dopamine molecules as robust anchoring agents to the iron-oxide core. The GM3-IONPs were fully characterized through various techniques and their stability in the cell culture medium DMEM was investigated. In the presence of a high salt and high protein environment of DMEM and FBS, IONPs were able to form a nanoparticle-corona layer on their surface within a rapid time duration. The size diameter of PEO-IONPs increased significantly compared to GM3-IONPs, suggesting the formation of thick nanoparticle-corona layer. PEO-IONPs were able to cause a significant decrease in the cell viability of CCD-18Co cells at concentrations above 100 μg/mL (IC50 = 68.025 μg/mL, Figure S9), whereas GM3-IONPs did not show significant cytotoxic effects on the cells (IC50 is greater than 500 μg/mL, Figure S9). Additionally, intracellular ATP levels of CCD-18Co were significantly diminished in the presence of PEO-IONPs but not GM3-IONPs, indicating interference in the activity of the ATP synthase pump by PEO-IONPs. Qualitative observations suggest that PEO-IONPs compromised the membrane integrity of the cells; however, cells exposed to GM3-IONPs showed significantly different cell morphology but no apparent membrane damage, which indicates subtle changes in the cytoskeleton arrangement of the cells. In the presence of PEO-IONPs or GM3-IONPs, cells exhibited a substantial decrease in their intracellular GSH levels in a time- and concentration-dependent manner that clearly denotes the existence of increased oxidative stress via the formation of ROS. This observation is supported in the DHR-123 assay, which indicated an increased amount of ROS from the control group after exposure to either PEO- or GM3-IONPs (Figure S8). Finally, the study indicates that IONPs were able to induce apoptosis in normal colon cells by means of measuring the activity of caspase proteins. The levels of caspase3 and caspase7 proteins were found to be significantly elevated in the cells in the presence of PEO-IONPs at higher concentrations, which was dependent on the exposure time. Based on the results that were obtained, it can be appropriately assumed that the reduction of GSH and oxidation of DHR-123 are from increased ROS levels, increased production of caspase3/7 proteins leads to apoptosis, and presumed IONPs uptake are a few of the prominent factors responsible for triggering cellular toxicity in CCD-18Co cells in the presence of PEO-IONPs.

There are some important nanoparticle synthesis parameters that directly affect the toxic potential of IONPs and warrant further in-depth studies. First, controlling the amount of PEO-PAA polymer groups that are grafted onto the surface of IONPs as excessive free groups can interact with protein molecules, altering the physio-chemical properties of IONPs. It is important to mention here that the presence of glycoconjugate molecules on IONPs renders them relatively biocompatible and efforts should be made to increase the general efficiency of ‘click chemistry’ reactions so that more glycoconjugate molecules are being attached to PEO-PAA polymer. Another important aspect to be considered is the overall concentration of IONPs used. In this study, the doses of IONPs used were high, meaning that if this were to be applied to a clinical setting for treatment, care must be taken to ensure that the dosage would not potentially damage the liver from iron toxicity as the IONPs are broken down by metabolic processes. Furthermore, investigation of the immunotoxicity of IONPs is necessary as colon cells can trigger inflammatory response signaling pathways in the presence of IONPs, leading to apoptosis. The reduction in GSH levels of colon cells in the presence of IONPs was observed, implying an increase in ROS generation. Gene expression studies of the ROS gene cluster would be valuable to explain the exact underlying mechanisms of increased oxidative stress that could also lead to apoptosis. By attaching proper antioxidant chemicals/drugs to GM3-IONPs, they could be effectively used for targeted drug delivery to colon cells remotely via magnetothermal drug release mechanisms in the presence of alternate magnetic fields for therapeutic applications in treating infections caused during post-IBS.

## Figures and Tables

**Table 1:** Dynamic light scattering measurements—Hydrodynamic diameter of IONPs as measured by dynamic light scattering in presence of cell-culture medium DMEM.

Hydrodynamic Diameter Z. Average (nm) of IONPs in Presence of Cell-Culture Medium for Different Time-Intervals						
Time	PEO-IONPs (in H_2_O)	PEO-IONPs + DMEM	PEO-IONPs + DMEM + 10% FBS	GM3-IONPs (in H_2_O)	GM3-IONPs + DMEM	GM3-IONPs + DMEM + 10% FBS
-	nm	nm	nm	nm	nm	nm
t = 5 min	78.8	173.0	167.0	88.8	104.0	91.1
t = 24 h	78.4	172.0	164.7	88.6	106.0	106.0
t = 48 h	78.1	171.2	165.8	88.1	106.8	109.9
t = 72 h	78.1	172.5	164.8	88.3	108.0	110.3

## Data Availability

The data presented in this study are available in this article and the supplementary material.

## Supplementary Materials

The following are available online at https://www.mdpi.com/article/10.3390/nano11102465/s1, Figure S1: Iron oxide nanoparticles as synthesized before functionalization with PEO-PAA-dopamine polymer, Figure S2: Functionalization steps of PEO-PAA-dopamine polymer with GM3, Figure S3: Moment vs. Field (MvH) loop showing the superparamagnetic behavior of the iron oxide nanoparticles, Figure S4: HNMR of the final PEO-PAA-dopamine, Figure S5: FTIR spectra of nanoparticles, Figure S6: Dynamic light scattering (DLS) intensity graphs for PEO-IONPs in presence of DMEM and 10% FBS, Figure S7: Dynamic light scattering (DLS) intensity graphs for GM3-IONPs in presence of DMEM and 10% FBS, Figure S8: Intracellular dihydrorhodamine (DHR-123) levels of CCD-18Co cells in the presence of IONPs, Figure S9: IC50 curves of nanoparticles against CCD-18Co cells, and Table S1: Dynamic light scattering and zeta-potential of IONPs.
