# Supplementary material for: Assessing the Biocompatibility of Multi-Anchored Glycoconjugate Functionalized Iron Oxide Nanoparticles in a Normal Human Colon Cell Line CCD-18Co
Source: Nanomaterials (Basel). 2021 Sep 22;11(10):2465. doi: 10.3390/nano11102465 (PMC8537094; doi:10.3390/nano11102465)
Supplement: Supplementary file 1 [file nanomaterials-11-02465-s001.zip › nanomaterials-1337612-supplementary.pdf]

nanomaterials

Supplementary Information

Assessing the Biocompatibility of Multi-Anchored Glycoconjugate Functionalized Iron Oxide Nanoparticles in a Normal Human Colon Cell Line CCD-18Co

Yash S. Raval [1], Anna Samstag [1], Cedric Taylor [1], Guohui Huang [1], Olin Thompson Mefford [2,*] and Tzuen-Rong Jeremy Tzeng [1,*]

1 Department of Biological Sciences, Clemson University, Clemson, SC 29634, USA; yraval@g.clemson.edu (Y.R.); afogert@g.clemson.edu (A.S.); cedrict@g.clemson.edu (C.T.); ghuang@g.clemson.edu (G.H.)
2 Department of Materials Science and Engineering, Clemson University, Clemson, SC 29634, USA
* Correspondence: mefford@clemson.edu (O.T.M.); tzuenrt@clemson.edu (T.-R.J.T.); Tel.: +1-864-656-4307 (O.T.M.); +1-864-650-8020 (T.-R.J.T.)

S.1 Iron Oxide Nanoparticle Synthesis and Functionalization

Iron oxide nanoparticles were synthesized using a one-pot thermal decomposition of iron (III) acetylacetonate (Sigma-Aldrich) and oleic acid (Alfa Aesar™, Fisher Scientific) [1,2]. Particles had an average diameter of 21.14 nm with a standard deviation of 2.16 nm (Figure S1). Polymer design was based on work by Stone et al. where a multi-anchored binding approach showed increased stability in comparison to polymer ligands with a single binding moiety (Figure S2) [3]. The moment vs. field (MvH) (Figure S3) measurement was done on the particles to confirm the super-paramagnetic behavior of the IONPs.

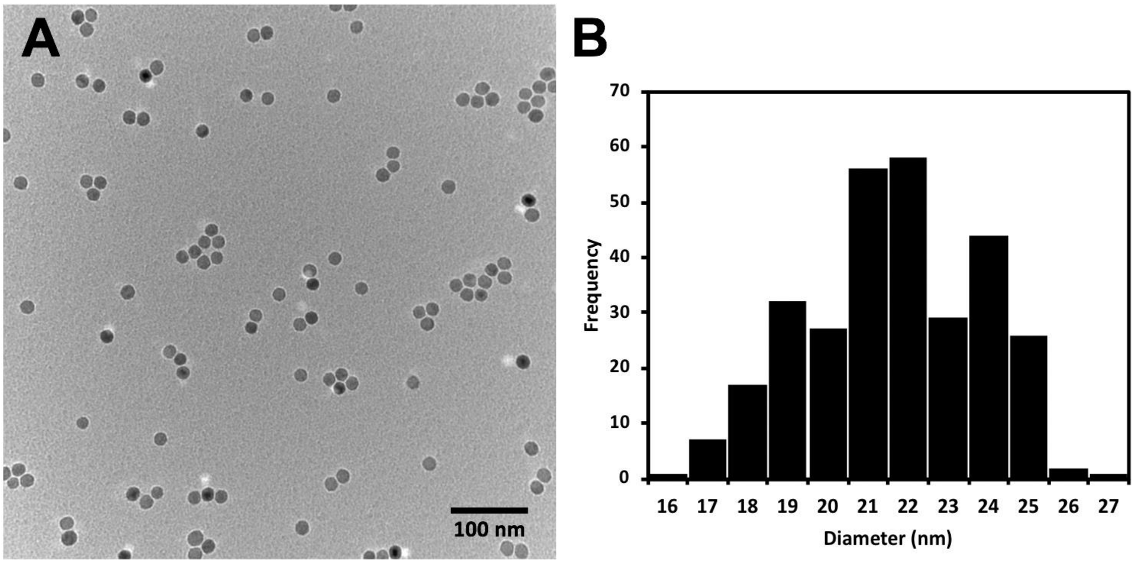

Figure S1. Iron oxide nanoparticles as synthesized before functionalization with PEO-PAA-dopamine polymer. A – Representative TEM image of the particles. B – Histogram depicting the particle size distribution.

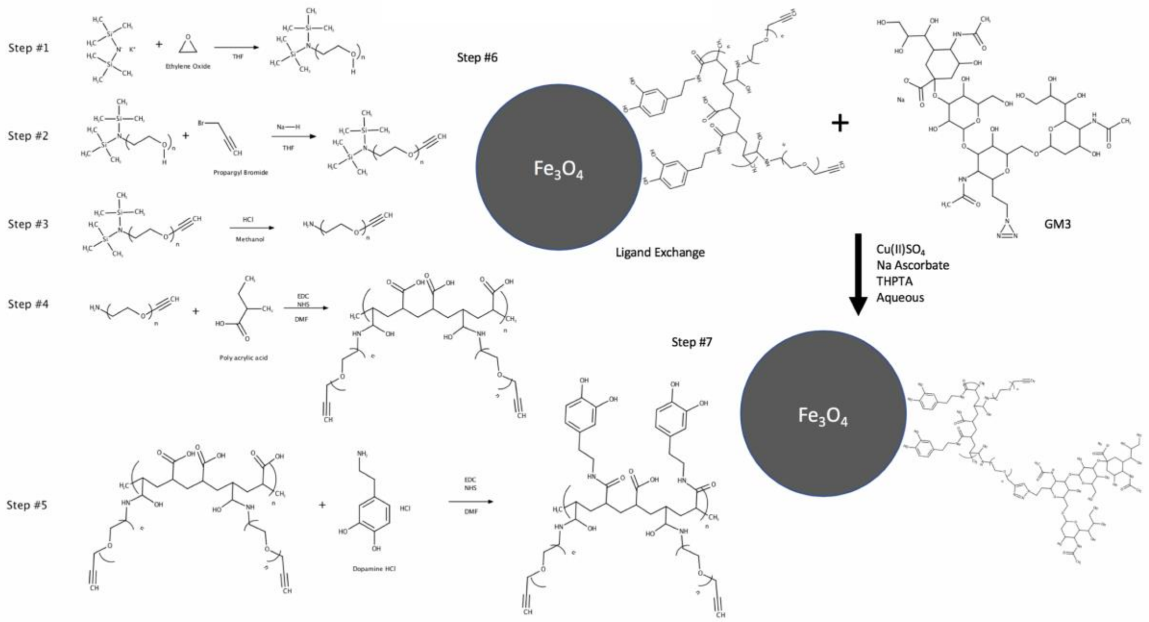

Figure S2. Functionalization steps of PEO-PAA-dopamine polymer with GM3. Left: 1) Anionic ring opening of ethylene oxide, 2) alkyne functionalization with propargyl bromide, 3) deprotection of primary amine, 4) coupling of PEO to PAA, 5) coupling of dopamine hydrochloride to the PEO-PAA. 6-7) Right: Click reaction between polymer coated particles and GM3 molecule.

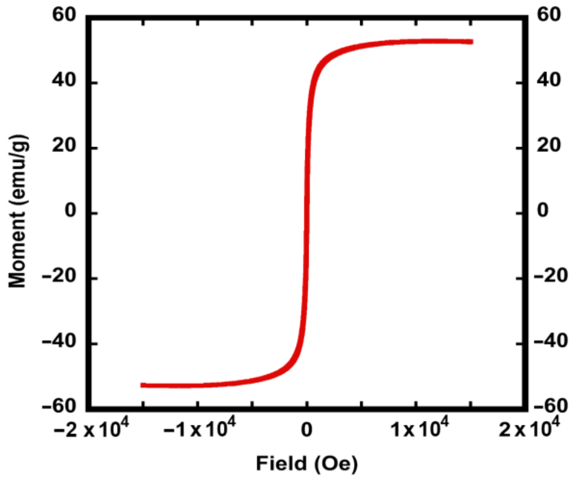

Figure S3. Moment vs. Field (MvH) loop showing the superparamagnetic behavior of the iron oxide nanoparticles (M_{sat} ~ 53 emu/g Fe). Figure adapted with permission from Raval et al. (2017) [4]; copyright 2021 John Wiley and Sons.

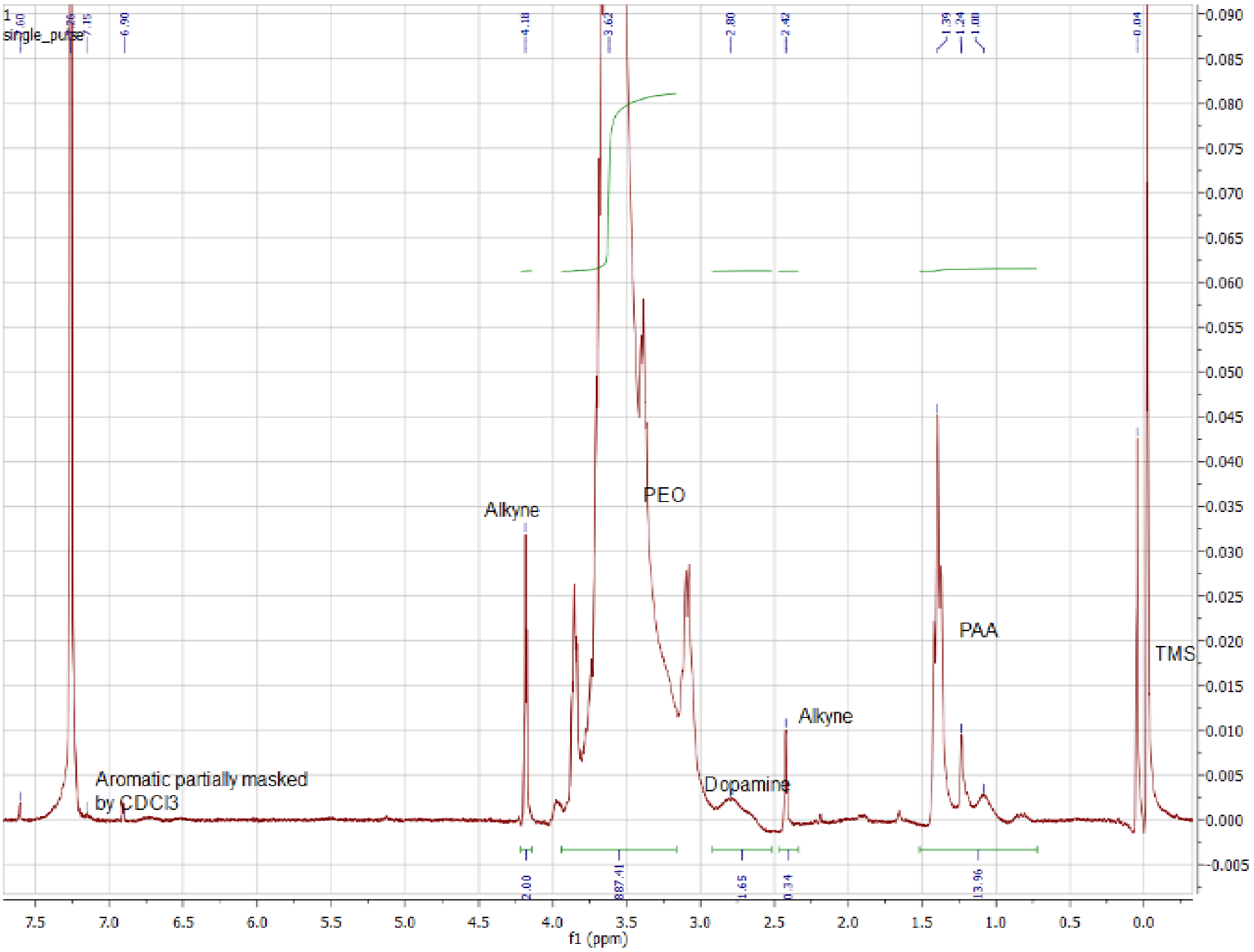

Figure S4. HNMR of the final PEO-PAA-dopamine - PAA backbone protons at 1.39 ppm - 1.08 ppm. Alkyne protons at 4.18 ppm and 2.42 ppm. PEO repeat protons at 3.62 ppm. Dopamine aromatic proton signal partially masked by the $CDCl_3$ peak, with alkane protons showing up at 2.8 ppm. Reference was tetramethylsilane at ~0 ppm. Figure adapted with permission from Raval et al. (2017) [4]; copyright 2021 John Wiley and Sons.

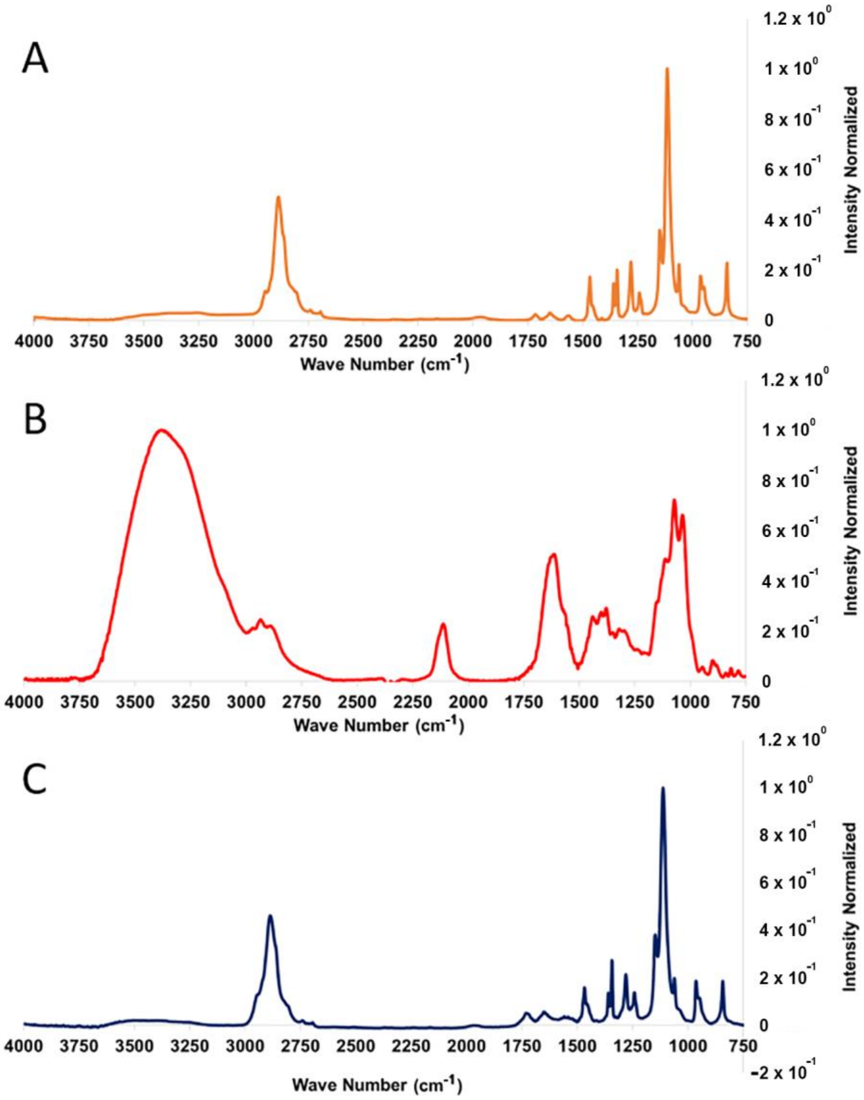

Figure S5. FTIR spectra of nanoparticles before GM3 conjugation (A), GM3 molecule (B), and after conjugation (C). The lack of the azide peak in C at 2100 cm^{-1} indicates purification of unbound GM3 after conjugation was successful. Figure adapted with permission from Raval et al. (2017) [4]; copyright 2021 John Wiley and Sons.

Table S1. Dynamic light scattering and zeta-potential measurements - Hydrodynamic diameter and zeta potential as measured by dynamic light scattering before and after GM3 conjugation. The increase in the hydrodynamic diameter indicates the GM3 glycoconjugate was successfully coupled to the PEO-IONPs.

	Hydrodynamic Diameter Z Avg. (nm) def	Zeta-potential (mV)
PEO-IONPs	114.4	−8.73
GM3-IONPs	120.3	−7.68

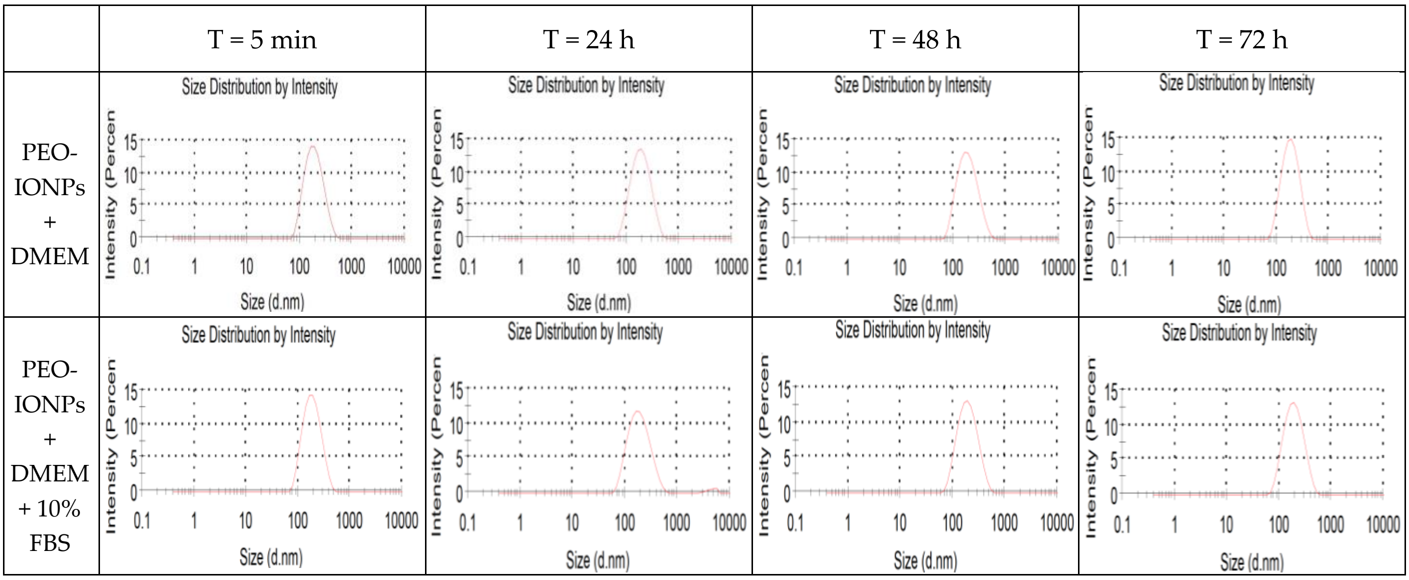

Figure S6. Dynamic light scattering (DLS) intensity graphs for PEO-IONPs in the presence of DMEM and 10% FBS.

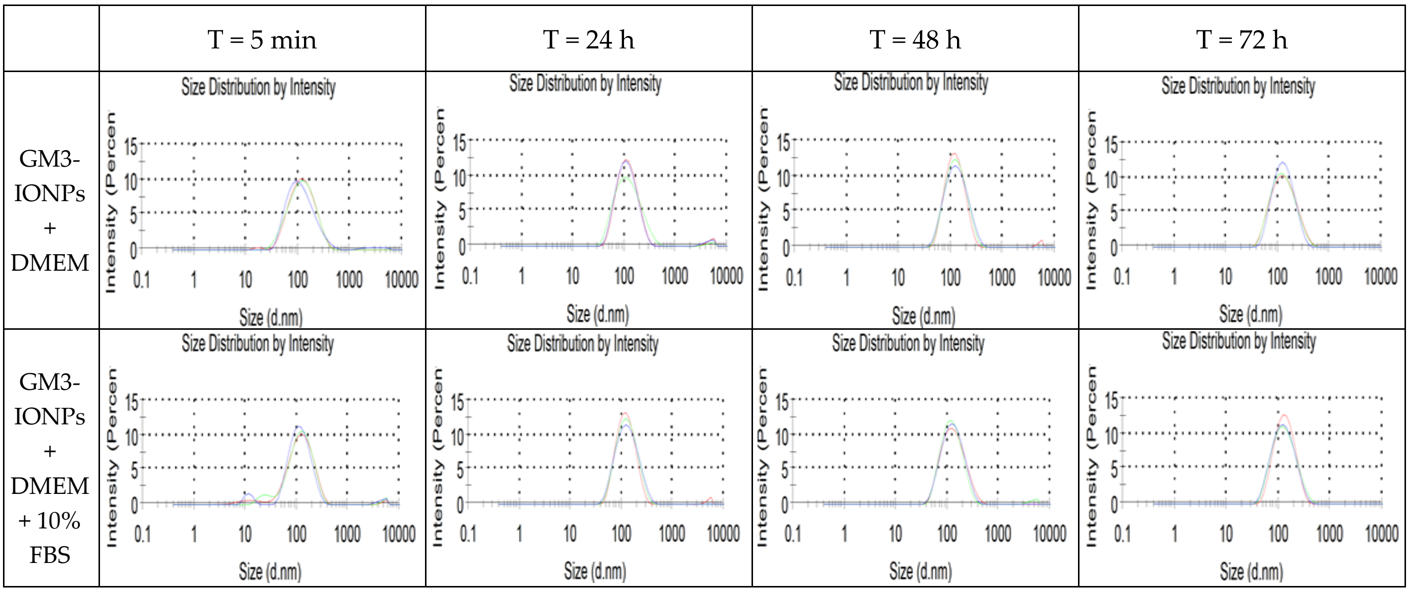

Figure S7. Dynamic light scattering (DLS) intensity graphs for GM3-IONPs in the presence of DMEM and 10% FBS.

S.2 Dihydrorhodamine-123 Assay of ROS in CCD-18Co Cells

Dihydrorhodamine-123 can serve as an indicator for the presence of intracellular ROS [5]. In the presence of ROS, the DHR-123 is oxidized to fluorescent rhodamine-123. This fluorescence can be measured, and the relative abundance of fluorescence indicates the oxidative stress levels of the cells. We measured the oxidized DHR-123 fluorescence of CCD-18Co cells treated with both PEO-IONPs and GM3-IONPs in order to understand the influence that either IONPs have on the production of ROS [6]. From our results (Figure S8) we observed that after 24 hours of incubation, the level of oxidized DHR-123 is increased significantly in the PEO-IONPs group from concentrations of 50 μg/mL – 500 μg/mL. Additionally, the GM3-IONPs group treated with 500 μg/mL showed significant deviation from the control group. In the 48 h treatment, the PEO-IONPs group showed a significant increase in the 100 μg/mL – 500 μg/mL group whereas the GM3-IONPs did not. Overall, the 48 h group had a lower fluorescence increase than the 24 h group, indicating the potential for a time dependent production of ROS when treated with IONPs.

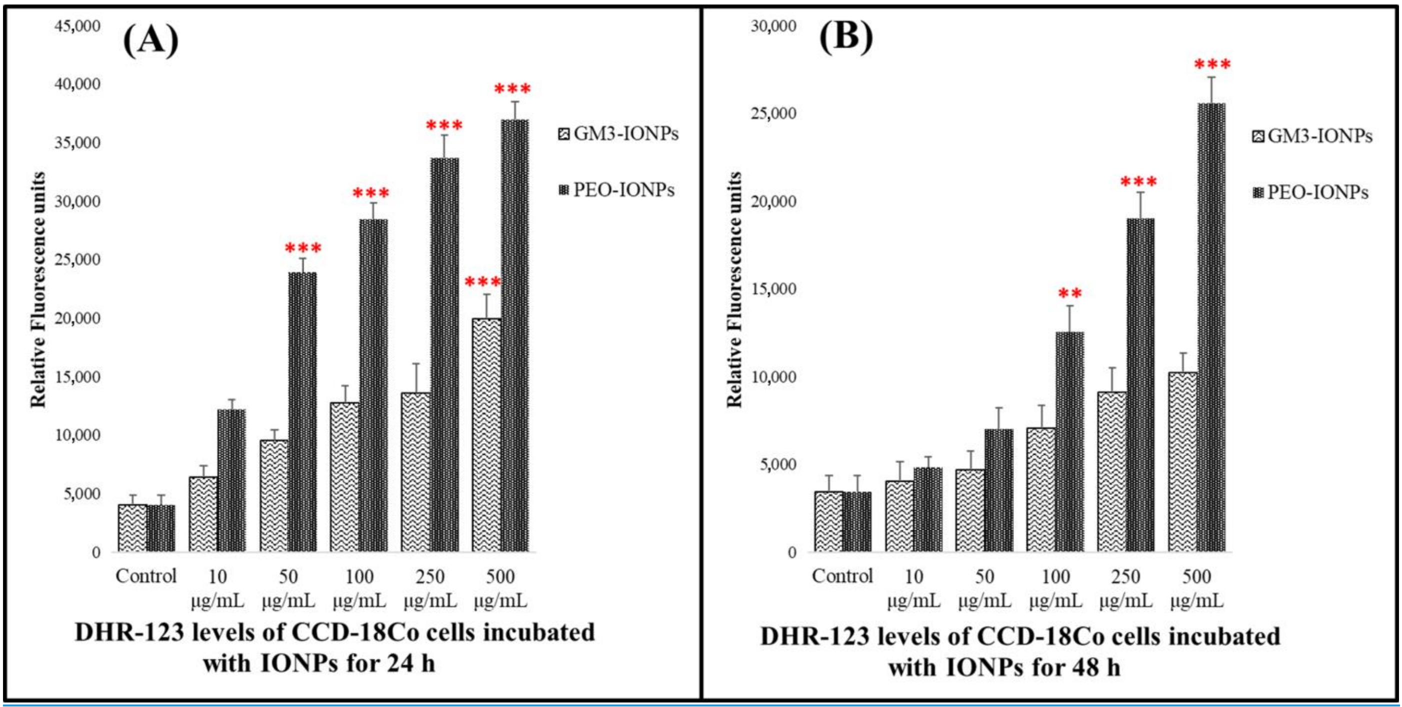

Figure S8. Intracellular dihydrorhodamine (DHR-123) levels of CCD-18Co cells in the presence of IONPs. A) ROS levels of cells exposed to PEO-IONPs at increasing concentrations for 24 h; B) ROS levels of cells exposed to GM3-IONPs at increasing concentrations for 48 h. Note the difference in the y-axis units. Data is expressed as Mean ± SD (n = 3); Statistical analysis – Analysis of Variance (ANOVA); ** *p*-value <0.01, and *** *p*-value <0.001.

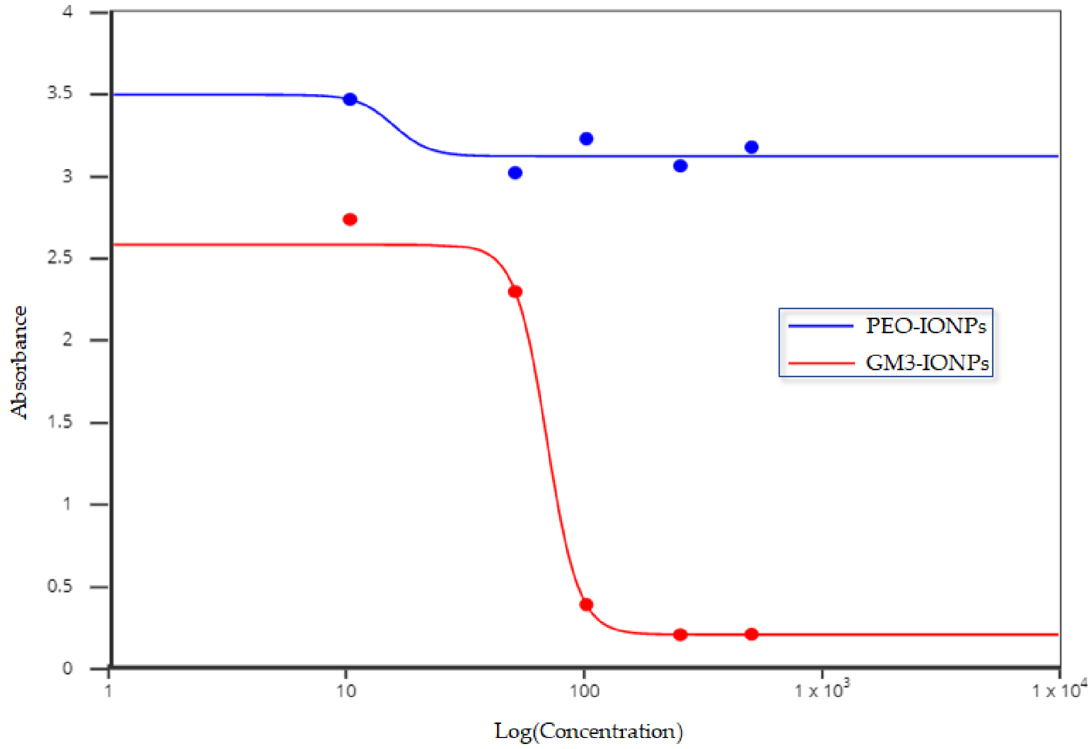

Figure S9. IC50 curves of PEO-IONPs and GM3-IONPs against CCD-18Co cells. The IC50 of PEO-IONPs is 68.025 µg/mL. The IC50 of GM3-IONPs is greater than 500 µg/mL, the maximum concentration tested. The IC50 was determined using the Quest Graph™ IC50 Calculator [7].

References

1. Vreeland, E.C.; Watt, J.; Schober, G.B.; Hance, B.G.; Austin, M.J.; Price, A.D.; Fellows, B.D.; Monson, T.C.; Hudak, N.S.; Maldonado-Camargo, L.; et al. Enhanced Nanoparticle Size Control by Extending LaMer's Mechanism. *Chem. Mater.* **2015**, *27*, 6059–6066, https://doi.org/10.1021/acs.chemmater.5b02510.
2. Sun, S.; Zeng, H.; Robinson, D.; Raoux, S.; Rice, P.M.; Wang, S.X.; Li, G. Monodisperse MFe2O4(M = Fe, Co, Mn) Nanoparticles. *J. Am. Chem. Soc.* **2004**, *126*, 273–279, https://doi.org/10.1021/ja0380852.
3. Stone, R.; Fellows, B.; Qi, B.; Trebatoski, D.; Jenkins, B.; Raval, Y.; Tzeng, T.-R.; Bruce, T.; McNealy, T.; Austin, M.; et al. Highly stable multi-anchored magnetic nanoparticles for optical imaging within biofilms. *J. Colloid Interface Sci.* **2015**, *459*, 175–182, https://doi.org/10.1016/j.jcis.2015.08.012.
4. Raval, Y.S.; Fellows, B.D.; Murbach, J.; Cordeau, Y.; Mefford, O.T.; Tzeng, T.J. Multianchored Glycoconjugate-Functionalized Magnetic Nanoparticles: A Tool for Selective Killing of Targeted Bacteria via Alternating Magnetic Fields. *Adv. Funct. Mater.* **2017**, *27*, https://doi.org/10.1002/adfm.201701473.
5. Qin, Y.; Lu, M.; Gong, X. Dihydrorhodamine 123 is superior to 2,7-dichlorodihydrofluorescein diacetate and dihydrorhodamine 6G in detecting intracellular hydrogen peroxide in tumor cells. *Cell Biol. Int.* **2008**, *32*, 224–228, https://doi.org/10.1016/j.cellbi.2007.08.028.
6. Do, M.; Stinson, K.; George, R. Reflectance structured illumination imaging of internalized cerium oxide nanoparticles modulating dose-dependent reactive oxygen species in breast cancer cells. *Biochem. Biophys. Rep.* **2020**, *22*, 100745, https://doi.org/10.1016/j.bbrep.2020.100745.
7. Quest Graph™ IC50 Calculator." AAT Bioquest, Inc, 25 Aug. 2021, https://www.aatbio.com/tools/ic50-calculator.
